# Association of depression phenotypes and antidepressant treatment with mortality due to cancer and other causes: a community-based cohort study

**DOI:** 10.3389/fpsyg.2023.1192462

**Published:** 2023-08-24

**Authors:** Anna Vilalta-Lacarra, Joan Vilalta-Franch, Domènec Serrano-Sarbosa, Ruth Martí-Lluch, Jaume Marrugat, Josep Garre-Olmo

**Affiliations:** ^1^Department of Medical Oncology, Clinica Universidad de Navarra, Pamplona, Spain; ^2^Girona Biomedical Research Institute (IDIBGI), Girona, Spain; ^3^Institut d'Assistencia Sanitaria, Girona, Spain; ^4^Department of Medical Sciences, University of Girona, Girona, Spain; ^5^Vascular Health Research Group (ISV-Girona), Foundation University Institute for Primary Health Care Research Jordi Gol i Gurina, Girona, Spain; ^6^IMIM-Institut Hospital del Mar d'Investigacions Mèdiques, Barcelona, Spain; ^7^CIBERCV de Investigación en Enfermedades Cardiovasculares, Instituto de Salud Carlos III, Madrid, Spain; ^8^Department of Nursing, University of Girona, Girona, Spain

**Keywords:** depressive syndrome, somatic symptoms, mortality, antidepressant drug, cancer

## Abstract

**Objective:**

This study aimed to assess the association of somatic depressive symptoms (SDS), cognitive/emotional depressive symptoms (C-EDS), and antidepressant treatment on mortality due to cancer and other causes in a community cohort.

**Methods:**

A community-based sample recruited in 1995, 2000, and 2005 aged between 35 and 75 years was examined in two waves and followed for a median of 6.7 years. SDS and C-EDS phenotypes were assessed using the Patient Health Questionnaire-9. Medication used by participants was collected. Deaths and their causes were registered during follow-up. Cox proportional hazard models stratified by sex were performed to determine the association between depressive phenotypes and mortality.

**Results:**

The cohort consisted of 5,646 individuals (53.9% women) with a mean age of 64 years (SD = 11.89). During the follow-up, 392 deaths were recorded, of which 27.8% were due to cancer. C-EDS phenotype was associated with an increased risk of cancer mortality in both men (HR = 2.23; 95% CI = 1.11–4.44) and women (HR = 3.69; 95% CI = 1.69–8.09), and SDS was significantly associated with non-cancer mortality in men (HR = 2.16; 95 CI % = 1.46–3.18). Selective serotonin reuptake inhibitors (SSRIs) were significantly associated with both cancer (HR = 2.78; 95% CI = 1.10–6.98) and non-cancer mortality (HR = 2.94; 95% CI = 1.76–4.90) only in the male population.

**Conclusion:**

C-EDS phenotype was related to an increased risk of cancer mortality at 6 years. In addition, the use of SSRIs in the male population was associated with cancer and all-cause mortality.

## 1. Introduction

Mental health disorders (Lu et al., 2016; Mallet et al., 2018), including depressive disorders (Mitchell et al., 2011; Lu et al., 2016), are highly prevalent in cancer patients throughout the disease course. The occurrence of depressive symptoms in cancer patients surpasses that of the general population (Mitchell et al., 2011), ranging from 8 to 24% (Krebber et al., 2014) depending on age (Akechi et al., 2020), sex (Chang et al., 2015), education level (Akechi et al., 2012; Zhang et al., 2021), cancer type (Massie, 2004; Dalton et al., 2009; Walker et al., 2014; Jia et al., 2017), anticancer treatment received (Caruso and Breitbart, 2020), depression intensity (Chang et al., 2015), and depression diagnosis tools used (Walker et al., 2013; Krebber et al., 2014; Caruso et al., 2017). The association between depression and cancer is particularly high during the first year after the cancer diagnosis, which then gradually decreases over time (Dalton et al., 2009; Lu et al., 2016). Indeed, increased rates of depression diagnosis are already observed in the year preceding the cancer diagnosis (Dalton et al., 2009) and even 5 years prior to cancer detection (Lu et al., 2020). Cancer patients frequently experience a complex set of physical and psychological symptoms that significantly impact their quality of life. The frequency of depressive symptoms and pain can explain the high consumption of antidepressants, including selective serotonin reuptake inhibitors (SSRI) (Zou and Zhu, 2022). Although some epidemiological studies support the association of SSRIs with an increased risk of mortality, at least in certain types of cancer (Fischer et al., 2022), the biological evidence is not conclusive (Stapel et al., 2021).

Depression is a heterogeneous syndrome in symptom profiling (Fried and Nesse, 2015), disease outcomes, and treatment responses which hinder our understanding of its etiology (Rush et al., 2004; Goldberg, 2011; Flint and Kendler, 2014; Krebber et al., 2014; Milaneschi et al., 2020). A depression phenotype with somatic/neurovegetative symptoms has been repeatedly observed and consistently linked to a wide set of demographic and clinical variables (Lux and Kendler, 2010; Leavens et al., 2012). This phenotype is associated with a more persistent disease course (Wa et al., 2019) and a poorer prognosis (Bekhuis et al., 2016), particularly among patients with heart disease (de Miranda Azevedo et al., 2014), leading to increased mortality and suicide risk (Jeon et al., 2016). The relationship between somatic symptoms and sex is not fully understood, as some studies have reported increased somatic symptoms (Wenzel et al., 2005; Jeon et al., 2016) and a higher prevalence of somatic depression in women (Silverstein, 2002), while others have registered more somatic symptoms in men (Castellanos et al., 2020). In contrast to cognitive/emotional symptoms of depression, somatic symptoms are less commonly observed at the onset of depression disease and increase with its evolution (Wa et al., 2019).

Although there are recommendations for using depression phenotypes in both clinical practice (Malhi and Mann, 2018) and research (Fried and Nesse, 2015) to assess cancer risk, they have rarely been considered to predict mortality risk. This study aimed to investigate the impact of the somatic and cognitive/emotional depression phenotypes on the risk of mortality from cancer and other causes in a community sample. Furthermore, the study aimed to explore the effects of antidepressant treatment on the different causes of mortality.

## 2. Materials and methods

### 2.1. Study design and setting

This was a cohort study that used data from the fourth and fifth follow-up assessments of the Regicor Study, a prospective population-based study about cardiovascular risk factors (http://www.regicor.org). The Regicor Study includes three representative population samples of Girona Province (Catalonia, Spain) recruited in 1995, 2000, and 2005.

### 2.2. Participants and sampling

The recruitment details have been previously described (Grau et al., 2007). In summary, individuals living in the city of Girona and three surrounding villages were randomly selected from the census and invited to participate. At recruitment, participants were aged 35–79 years, had lived in the referral area for at least 6 months, had no terminal diseases, and were not institutionalized. Between May 2017 and October 2019, 6,529 participants of the cohort were contacted again to perform the clinical follow-up, of which 5,646 participants responded (response rate of 86.47%).

### 2.3. Data collection and measures

Data collection was organized in the Primary Care settings of the study participants' towns. Participants were informed by postal mail and received a telephone call to schedule the examination by telephone call. The examination was standardized with all the participants being examined in the same order.

The clinical interview included sociodemographic information and the completion of health questionnaires. Depressive symptoms were assessed using the Patient Health Questionnaire (PHQ-9), a 9-item depression screening tool that evaluates the frequency of depressive symptoms over the past 2 weeks (Kroenke et al., 2001). The nine items were selected based on the nine criteria evaluated by the DSM IV for major depression with scores ranging from 0 to 27. The PHQ-9 is a well-established validated tool with a sensitivity of 80–90% for major depression screening and is highly recommended in clinical practice (Levis et al., 2019; Zimmerman, 2019).

Depressive symptoms were defined by a score punctuation of ≥5 points, including all mild, moderate, and severe symptoms. In addition, two subscales were considered: one with somatic symptoms (sleep disorders, fatigue, appetite disturbances, and psychomotor agitation or retardation) (de Jonge et al., 2007); and the second with the remaining five cognitive/emotional items (depressed mood, diminished interest in most activities, feelings of worthlessness or inappropriate guilt, diminished ability to think, concentrate or indecisiveness, and suicidal ideation or thoughts of death). Participants were categorized as presenting somatic depressive symptoms (SDS) if they had a PHQ-9 ≥ 5 and the somatic subscale score was higher than that obtained in the cognitive/emotional one. If the cognitive/emotional subscale score was superior to the somatic subscale, participants were categorized as presenting cognitive/emotional depressive symptoms (C-EDS).

The following risk factors were measured in the fourth follow-up assessment using standardized methods based on the World Health Organization recommendations (Tunstall-Pedoe et al., 1994). Using a standardized questionnaire on smoking, participants were classified as smokers (current or quit < 1 year) or non-smokers (quit ≥ 1 year or never smoked). Systolic and diastolic blood pressure and resting heart rate (RHR) were measured with calibrated sphygmomanometers under standardized conditions. Fasting blood samples were taken and glucose, total cholesterol, high-density lipoprotein (HDL) cholesterol, low-density lipoprotein (LDL) cholesterol, and triglyceride concentrations were determined. Hypertension was defined as systolic blood pressure (SBP) ≥ 140 mmHg, diastolic blood pressure (DBP) ≥ 90 mmHg, or participants on antihypertensive treatment; diabetes as fasting glucose > 125 mg/dL or participants already on antidiabetic treatment; and dyslipidemia as HDL < 40 mg/dl in men or HDL < 50 mg/dl in women, or LDL > 159 mg/ml or patients already under treatment (Expert Panel on Detection, Evaluation, and Treatment of High Blood Cholesterol in Adults, 2001).

High waist circumference was established at >102 cm in men and >88 cm in women. The bilateral ankle–brachial index was calculated as the ratio of the systolic pressure at the ankle to the systolic pressure at the arm (Hirsch et al., 2006). Peripheral artery disease was considered when registered in medical records or the ankle–brachial index was < 0.9 regardless of claudication symptoms (ACCF/AHA members, 2011). In addition, a history of coronary heart disease or stroke was registered from the electronic medical records of the study participants.

An atherosclerotic and cardiovascular burden index (ACVb index) (range 0 to 9 points) was calculated according to the presence of a personal history of stroke, coronary disease, peripheral arterial disease, diabetes, hypertension, dyslipidemia, smoking status, high waist circumference, and family history of arteriosclerotic disease. In addition, participants under treatment with inhaled corticosteroids, bronchodilators, inhaled anticholinergics, or antileukotrienes were considered as having a chronic obstructive pulmonary disease (COPD).

Psychopharmacological treatment of participants was registered at the fourth follow-up assessment and classified according to the Anatomical, Therapeutic, and Chemical classification System: SSRIs [ATC-N06AB], other antidepressant treatments [all drugs from section N06AX], and anxiolytic and hypnotic treatments corresponding to drugs included in sections N05B and N05C.

### 2.4. Mortality ascertainment

Vital status and cause of death during the follow-up were ascertained by examining the corresponding electronic medical records for in-hospital deaths and reviewing death certificates from regional and national registers for out-of-hospital deaths. All deaths were encoded according to the ICD-10. Mortality was classified as being due to all malignant neoplasms (ICD C00–C99 and D1–D48) or other diseases (the rest of the ICD codes).

### 2.5. Statistical analysis

Standard parametric and non-parametric tests were used to compare baseline characteristics of participants who died from cancer, those who died from other causes, and survivors. To assess the magnitude of the effect, Cohen's *d* (d) was used for continuous variables and Cramer's V (V) for categorical ones. We estimated mortality rates using the person-years method, and 95% confidence intervals (CIs) for mortality rates were calculated assuming a Poisson distribution for the number of deceased subjects. Cancer mortality Kaplan–Meier survival curves and Breslow tests (predominance of events at early follow-up) were computed for both SDS and C-EDS. The association between depressive symptoms phenotypes and mortality from cancer and other causes was assessed using a Cox Proportional-Hazard model stratified by sex. The model was controlled by statistically significant variables identified in the bivariate analyses.

The results are expressed as absolute numbers and percentages, mean or median, standard deviation (SD) or interquartile range (IQR), effect size measures, hazard ratios, and 95% CIs. All statistical analyses were conducted using the IBM SPSS Statistical package for Windows version 22.0, and we employed an alpha level for statistical significance of 0.05 (two-tailed).

## 3. Results

### 3.1. Characteristics of study participants

The sample consisted of 5,646 participants, of which 53.9% were women, with a mean age of 61 years old (SD = 12; range = 35–95). Table 1 reports demographic and clinical characteristics stratified according to the vital status (dead due to cancer or other causes) and surviving participants.

**Table 1:** Demographic and clinical characteristics among dead (cancer and other causes) and surviving participants.

	**Cancer (a) (*n* = 109)**	**Other (b) (*n* = 283)**	**Survivors (c) (*n* = 5,254)**	**Effect size (a vs. c)**	**Effect size (b vs. c)**
Women, *n* (%)	35 (32.1)	103 (36.4)	2,907 (55.3)	**0.066** ^**†**^	**0.084** ^**†**^
Age, mean (SD)	69.3 (10.3)	76.7 (8.4)	60.2 (11.4)	**0.791** ^**‡**^	**1.453** ^**‡**^
**Marital status**, ***n*** **(%)**					
Married	84 (77.1)	178 (62.9)	4,076 (77.8)	0.005^**†**^	**0.121** ^**†**^
Single	13 (11.9)	29 (10.2)	642 (12.3)		
Widow	12 (11.0)	178 (62.9)	519 (9.9)		
**Educational level**, ***n*** **(%)**					
Low	59 (54.1)	201 (73.7)	2,647 (50.9)	0.019^**†**^	**0.098** ^**†**^
Average	25 (22.9)	40 (14.6)	1,504 (28.9)		
High	25 (22.9)	33 (12.0)	1,049 (20.2)		
**Employment status**, ***n*** **(%)**					
Active/household chores	38 (34.9)	52 (18.6)	3,122 (59.8)	**0.076** ^**†**^	**0.209** ^**†**^
Retired	64 (58.7)	222 (79.3)	1,757 (33.7)		
Unemployed/others	7 (6.4)	6 (2.1)	339 (6.5)		
Resting heart rate (≥80), *n* (%)	24 (22.0)	64 (22.6)	844 (16.1)	0.023^**†**^	**0.039** ^**†**^
COPD, *n* (%)	6 (5.5)	32 (11.3)	168 (3.2)	0.018^**†**^	**0.096** ^**†**^
Anxiolytics and hypnotics, *n* (%)	12 (11.0)	62 (21.9)	695 (13.2)	0.009^**†**^	**0.056** ^**†**^
SSRI, *n* (%)	10 (9,2)	28 (9.9)	402 (7.7)	0.008^**†**^	0.018^†^
Other antidepressants, *n* (%)	1 (0.9)	2 (0.7)	80 (1.5)	0.007^**†**^	0.015^†^
**Depression phenotype**, ***n*** **(%)**					
Cognitive/emotional	23 (21.9)	40 (14.7)	674 (13.0)	**0.040** ^**†**^	**0.040** ^**†**^
Somatic	16 (15.2)	51 (18.8)	674 (13.0)		
PHQ-9 (total score), mean (SD)	3.91 (3.8)	4.22 (4.3)	3.30 (4.0)	0.149^‡^	**0.224** ^**‡**^
ACVb index, mean (SD)	2.55 (1.3)	3.15 (1.4)	2.15 (1.3)	**0.369** ^**‡**^	**0.724** ^**‡**^
Resting heart rate, mean (SD)	71.6 (12.2)	71.3 (13.1)	69.5 (10.5)	**0.202** ^**‡**^	**0.175** ^**‡**^
Body mass index, mean (SD)	26.67 (3.7)	28.08 (5.0)	27.36 (4.5)	0.068^‡^	**0.157** ^**‡**^

COPD, Chronic obstructive pulmonary disease; SSRI, selective serotonin reuptake inhibitors; PHQ-9, Patient Health Questionnaire-9; ACVb, atherosclerotic and cardiovascular burden; ^†^χ^2^ for categorical variables; ^‡^Mann–Whitney U for numeric variables. Significant effect sizes in bold.

### 3.2. Epidemiology of cancer and non-cancer mortality

During the study follow-up, 392 deaths occurred, of which 109 (27.8%) were due to cancer. Overall crude mortality rates were 1,098, 540, and 803 per 100,000 inhabitants, and age-adjusted death rates using European standard population were 799 (95% CI = 545.8–1,052.2), 426 (95% CI 242–610), and 602 (95% CI 448–756) per 100,000 inhabitants for men, women, and overall, respectively. The cancer crude mortality rates of the study were 252, 112, and 175; and age-adjusted mortality rates were 178 (95% CI 59–297), 81 (95% CI 2.8–159), and 131 (95% CI 60–211) for men, women, and global, respectively. The median time to death from baseline was 4.32 years (IQR = 2.31).

Of all the individuals who died, 254 (64.8%) were men and 138 (35.2%) were women (*p* < 0.001). The baseline mean age of deceased participants was 74.6 years old (SD = 9.6) vs. 60.2 years old (SD = 11.3) of survivors (*p* < 0.001). Regarding participants who died from cancer, 67.9% were men and 32.1% were women (*p* < 0.001), with a mean age of 69.3 years (SD = 10.37) (*p* < 0.001). Of participants who died from non-cancer causes, 180 (63.6%) were men and 103 (36.4%) were women (*p* < 0.001) with a mean age of 76.7 years (SD = 8.47) (*p* < 0.001) (Table 1).

SDS and C-EDS phenotypes were observed in 15.2 and 21.9% of patients with cancer mortality (*p* = 0,040) and in 18.8 and 14.7% of patients that died from other causes (*p* = 0.012) compared with 13% and 13% of alive individuals, respectively (Table 1). Additionally, participants who died from cancer had a significantly higher resting heart rate (RHR) (71.69 bpm; SD = 12.20) compared with survivors (69.55 bpm; SD =10.59) (*p* = 0.037), and a higher ACVb index: 2.55 (SD = 1.38) vs. 2.15 (SD = 1.38), respectively (*p* < 0.001) (Table 1).

Besides, in participants with non-cancer deaths, a higher PHQ-9 score was reported compared with survivors: 4.22 points (SD = 4.36) vs. 3.30 points (SD = 4.09), respectively (*p* < 0.001); increased anxiolytic treatment (21.9 vs. 13.2 %; *p* < 0.001); higher ACVb index (3.15; U = 495,724.5; *p* < 0.001); and an increased BMI (28.08; SD = 5.07) (*p* < 0.01) (Table 1).

### 3.3. Depression phenotypes and mortality

**Figure 1:**
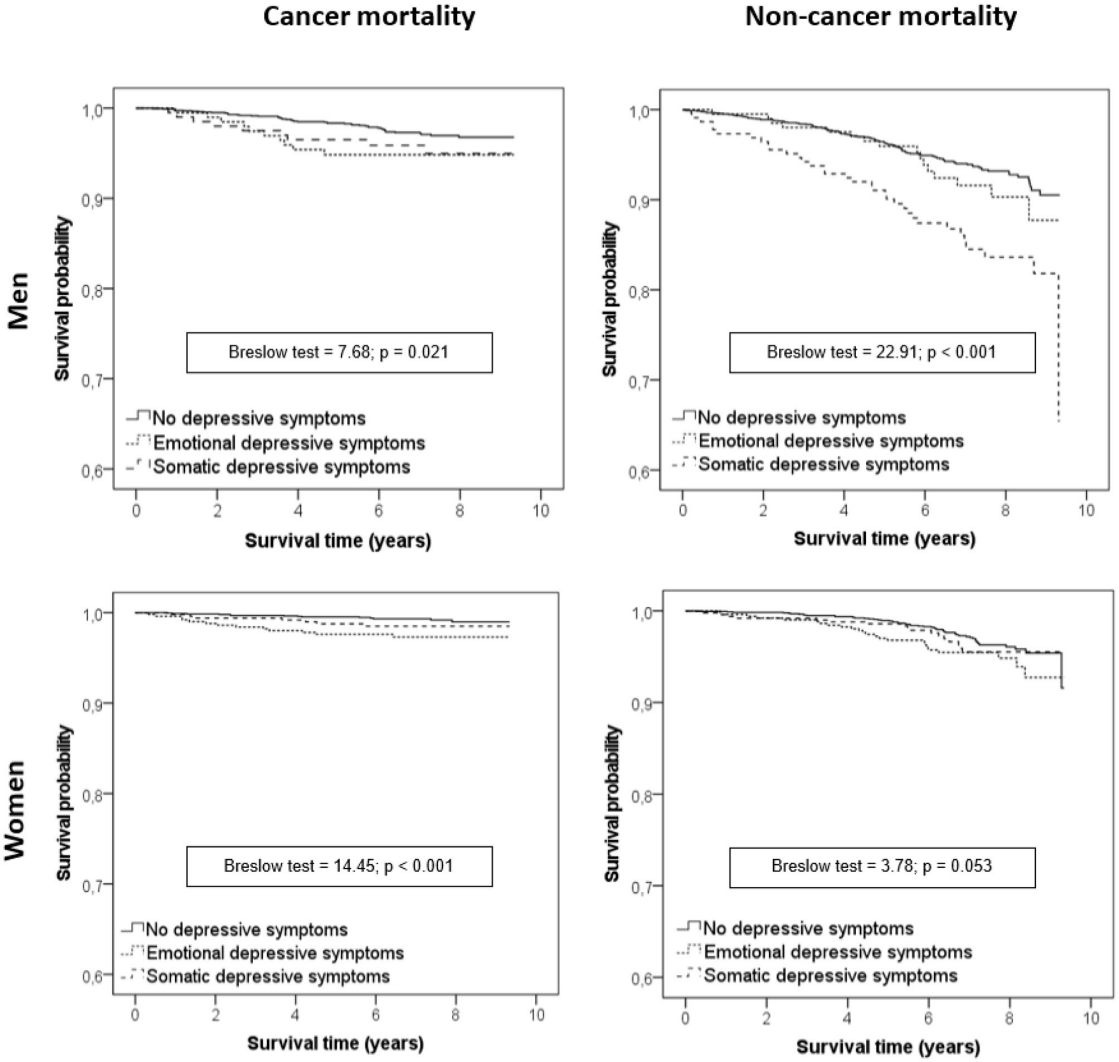
Kaplan–Meier survival curves according to depressive symptoms stratified by sex.

Significant differences were observed in cancer mortality survival curves according to depression phenotypes in both men (*p* = 0.021) and women (*p* < 0.001). Similarly, in non-cancer mortality survival curves, significant differences between depression phenotypes were also observed in both sexes (Figure 1). A Cox regression showed that C-EDS were significantly associated with cancer mortality in both men and women. Otherwise, no association was observed between SDS and cancer mortality. Regarding non-cancer mortality, SDSs were significantly associated only with men, with no association with C-EDS (Table 2).

**Table 2:** Variables related to mortality for cancer and for other causes stratified by sex.

	**Cancer mortality Hazard ratio (95% CI)**	**Other causes of mortality Hazard ratio (95% CI)**
**Men**	**(*****n*** = **71/2,354)**	**(*****n*** = **175/2,453)**
Age	**1.08 (1.05–1.11)**	**1.14 (1.12–1.16)**
ACVb index	1.14 (0.97–1.33)	**1.26 (1.13–1.39)**
COPD	1.29 (0.51–3.27)	**1.83 (1.15–2.91)**
Resting heart rate (≥80)	**1.99 (1.13–3.50)**	**1.84 (1.27–2.67)**
Anxiolytics and hypnotics	**0.30 (0.09–0.99)**	1.40 (0.94–2.09)
SSRI	**2.78 (1.10–6.98)**	**2.94 (1.76–4.90)**
Other antidepressants		0.19 (0.02–1.40)
**Depression phenotype**		
Cognitive/emotional	**2.23 (1.11–4.41)**	1.01 (0.59–1.72)
Somatic	1.95 (0.96–3.99)	**2.16 (1.46–3.18)**
**Women**	**(*****n*** = **34/2,853)**	**(*****n*** = **94/2,913)**
Age	**1.07 (1.039–1.120)**	**1.19 (1.15–1.23)**
ACVb index	1.01 0.78–1.32)	**1.39 (1.19–1.62)**
COPD	0.67 (0.09–5.03)	1.30 (0.58–1.52)
Resting heart rate (≥80)	1.27 (0.54–2.96)	1.27 (0.77–2.10)
Anxiolytics and hypnotics	0.97 (0.42–2.22)	0.94 (0.58–1.52)
SSRI	0.65 (0.22–1.95)	0.74 (0.38–1.47)
Other antidepressants	1.35 (0.17–10.36)	0.53 (0.07–3.90)
**Depression phenotype**		
Cognitive/emotional	**3.69 (1.69–8.09)**	1.17 (0.70–1.95)
Somatic	2.31 (0.92–5.80)	1.26 (0.71–2.23)

ACVb, atherosclerotic and cardiovascular burden; COPD, chronic obstructive pulmonary disease; SSRI, selective serotonin reuptake inhibitors. Statistically significant values in bold.

### 3.4. Other mortality risk factors

Additional risk factors for cancer mortality observed by Cox regression included age for both sexes and RHR and SSRI consumption only in men. On the other hand, anxiolytic treatment was associated with slightly lower risk. For non-cancer mortality, age and ACVb index were identified as risk factors in both men and women, while obstructive pulmonary disease, RHR ≥ 80 bpm, and consumption of SSRIs were significant mortality risk factors only in men (Table 2).

## 4. Discussion

We aimed to examine the association of depressive phenotypes and antidepressant treatment on the risk of mortality from cancer and other causes in a community-based sample. Our findings revealed that the cognitive-emotional depressive phenotype was associated with an increased risk of mortality for cancer in both men and women. The somatic depressive phenotype was found to increase mortality for other causes only in men. Besides, we observed that psychopharmacological treatments have a positive or negative effect on male cancer patients' survival depending on the type of treatment. Moreover, we identified several risk factors for non-cancer mortality in men that were not in women.

Somatic depressive symptoms have been associated with increased mortality (Colman et al., 2018). However, an overestimation of depressive disorders due to symptom overlap derived from somatic illnesses could partly explain this association (Rayner et al., 2011; Saracino et al., 2020). In cancer patients, SDSs are common (Del Piccolo et al., 2021), and being depressive, anxious, and fatigued are very specific depressive phenotypes in these patients (Schellekens et al., 2020). For depression screening in patients with cancer, somatic items showed a less discriminatory value than non-somatic ones, although they are still useful for mild/moderate depression detection, and ignoring them would lead to an underestimation of depressive syndromes (Grapp et al., 2019). Furthermore, the overestimation of depressive disorders at the expense of somatic symptoms among medically ill patients is scarce (Leavens et al., 2012). On the other hand, depression's affective and cognitive symptoms more robustly predicted long-term mortality in patients undergoing chronic hemodialysis (Cheng et al., 2018) and with chronic kidney disease (Kellerman et al., 2010). The results of the present study contribute that the C-EDS phenotype increases cancer mortality risk in both men and women, with a higher intensity in the latter. The C-EDS phenotype could be linked to an autonomic dysfunction that has been identified as a potential pathophysiological mechanism of depression (Valenza, 2023). In this sense, the heart rate measured in this study, which is regulated by the autonomic nervous system, can be interpreted as an indicator of autonomic dysfunction in men. Conversely, SDS phenotype is a risk factor for non-cancer mortality only in men, with no association between any depression phenotype and non-cancer mortality in women. These results suggest different biological mechanisms according to depression phenotype that could impact in terms of mortality.

The presence of depressive symptoms decreases treatment adherence in cancer patients (Hoogendoorn et al., 2019). In the study presented, there is no information about adherence to cancer treatment, which could bias our results as it prevents us from elucidating whether the cause of mortality is due to depressive symptomatology itself or a poor adherence to cancer treatment. However, in other chronic diseases, differences in treatment adherence between both dimensions of depressive symptoms have not been demonstrated (Theofilou and Panagiotaki, 2012). As our results only associate C-EDS with mortality from cancer, the authors consider the probability of such bias low.

The role of antidepressant treatment in cancer development remains controversial. SSRIs have shown potential anticancer properties by inhibiting cancer cell proliferation and inducing apoptosis (Amit et al., 2009). Berge et al. described an association between the use of antidepressants and a decreased risk of cutaneous melanoma (Berge et al., 2020), although the biological mechanism was not elucidated. On the other hand, prospective studies suggested an association between antidepressant treatment and some cancer types incidence such as breast cancer and colorectal cancer, which was not confirmed in tumor type-targeted studies (Kurdyak et al., 2002; Wu et al., 2015; Lin et al., 2016).

Epidemiological studies disclosed an association between antidepressant treatment and mortality risk for both cardiovascular and cancer causes (Aronow and Shamliyan, 2020; Fischer et al., 2022). Sun et al. reported a 30% increased cancer mortality risk during the first year after diagnosis, and up to 47% at 5 years depending on tumor type and antidepressant treatment (Sun et al., 2015). On the other hand, a beneficial effect of antidepressant treatment was observed in terms of lung cancer- specific survival, and norepinephrine reuptake inhibitors and tricyclic antidepressants were associated with improved survival (Zingone et al., 2017). However further studies did not demonstrate a survival benefit of antidepressant treatment neither in non-small-cell lung cancer, small-cell lung cancer (Nagla Fawzy et al., 2019), nor other neuroendocrine tumors (Riess et al., 2020). Our results confirm increased mortality for both cancer and non-cancer causes in men under SSRI treatment, but not in women, thus it is plausible to hypothesize that the physiopathogenic mechanism leading to increased mortality from SSRIs might be modulated by sex. Anxiolytic and hypnotic treatments have not registered a relevant increased mortality risk for any causes (Patorno et al., 2017). Existing evidence does not show an association between the use of benzodiazepines in cancer patients and decreased survival (O'Donnell et al., 2019). Our results suggest a protective effect of anxiolytics/hypnotics in male cancer patients. Most neuroprotective drugs are antioxidants, and benzodiazepines have been shown to have an antioxidant effect. The neuroprotective effect of low-dose benzodiazepines (Liu et al., 2016) and the improvement of some classical risk factors such as hypertension (Yeh et al., 2020) might explain our results. This neuroprotective effect could be attributed to its ability to mitigate chronic inflammation levels (Dominguini et al., 2021). Peripheral inflammation has been implicated in activating central immune–inflammatory pathways, thereby contributing to the development of pain, fatigue, and depressive symptoms in cancer patients (Borovcanin et al., 2023).

## 5. Limitations

Several limitations must be considered when interpreting our results. First, after stratification based on mortality cause and sex, some subgroups contained a scarce number of individuals with events that limited the statistical power, particularly among women. Second, lifestyle and dietary habits were not registered, and we were unable to clarify the magnitude of the effect on increased mortality that can be attributed to depressive symptomatology or as a consequence of lifestyle. Third, our results indicated an increased cancer mortality associated with cognitive-emotional depression phenotype; however, information regarding tumor type and treatment received was not available, and evidence suggests that this association might vary depending on primary tumor location (Wang et al., 2019). Fourth, the small number of participants with antidepressant treatment other than SSRIs did not allow us to report conclusive results with this type of drug. Fifth, the time of onset of depressive symptoms was not available, and the effect of symptoms' duration on mortality and whether depressive symptoms affect in the same way previous and after cancer diagnosis requires further investigation. Sixth, we were unable to control for the depressive symptomatology intensity, and we could not rule out a possible prescription bias. For instance, greater severity of depressive symptoms has been associated with more severe breast cancer treatment-related symptoms (Ganz et al., 2021). Seventh, the protective effect of anxiolytics/hypnotics observed should be considered cautiously because we did not have information regarding the dosage of these treatments.

## 6. Conclusion

Our results may have two important clinical implications. First to contribute to the use of the examination of depression phenotypes in clinical practice and second to consider individually the antidepressant treatment use in male and female cancer patients according to possible risks and benefits.

The cognitive-emotional depressive phenotype was found to be associated with an increased cancer mortality risk in both men and women. Additionally, it was associated with a higher non-mortality specifically in men. These findings suggest that different physiopathogenic mechanisms of depression phenotypes may influence mortality, and these mechanisms may be modulated by sex. However, it is also possible that the observed differences in mortality risks between men and women could be influenced by the statistical power of our study, with potentially lower statistical power in women. Regarding the use of selective serotonin reuptake inhibitors (SSRIs), our study indicated an increased mortality risk in men, regardless of the cause of death. Furthermore, we observed that other anxiolytics/hypnotics were associated with a lower risk of cancer mortality in men. However, further investigation is needed to fully understand the underlying mechanisms behind these associations. The observed differences in mortality risk factors according to sex also deserve further studies.

## Data availability statement

The raw data supporting the conclusions of this article will be made available by the authors, without undue reservation.

## Ethics statement

The requirement of ethical approval was waived by the Ethics Committee of the Parc de Salut MAR for the studies involving humans. The study was conducted in accordance with the local legislation and institutional requirements. Written informed consent was signed by all study participants.

## Author contributions

AV-L and JV-F conceptualized the study. JM and JG-O acquired funding. RM-L contributed to participant recruitment. JV-F performed the analysis of the data. The original draft was prepared by AV-L, JV-F, and JG-O. RM-L, JM, and DS-S critically reviewed the manuscript. All authors contributed to the article and approved the submitted version.
